# Identification of SETD4 as an Onco‐Immunological Biomarker Encompassing the Tumor Microenvironment, Prognoses, and Therapeutic Responses in Various Human Cancers

**DOI:** 10.1002/iid3.70126

**Published:** 2025-01-16

**Authors:** Yuyun Zhong, Ruiqi Wang, Zijie Huang, Zhaoting Hu, Bin Peng, Bin Chen, Liyue Sun

**Affiliations:** ^1^ Department of Health Management Center The Third Affiliated Hospital of Southern Medical University Guangzhou China; ^2^ The Guangzhou Bay Area Institute of Biomedicine Guangzhou China; ^3^ Department of Pharmacy, Zhuhai People's Hospital Zhuhai Hospital Affiliated With Jinan University Zhuhai China; ^4^ Department of Plastic Surgery, The First Affiliated Hospital of Jinan University Jinan University Guangzhou Guangdong China; ^5^ Department of Thoracic Surgery The First Affiliated Hospital of Southern University of Science and Technology, Shenzhen People's Hospital Shenzhen China; ^6^ Second Department of Oncology Guangdong Second Provincial General Hospital Guangzhou China

**Keywords:** cancer stemness, pancancer, prognosis, SETD4, tumor microenvironment

## Abstract

**Background:**

SET domain‐containing protein 4 (SETD4) is a histone methyltransferase that has been shown to modulate cell proliferation, differentiation, and inflammatory responses by regulating histone H4 trimethylation (H4K20me3). Previous reports have demonstrated its function in the quiescence of cancer stem cells as well as drug resistance in several cancers. A limited number of systematic studies have examined SETD4's role in the tumor microenvironment, pathogenesis, prognosis, and therapeutic response.

**Methods:**

Utilizing The Cancer Genome Atlas database, and other publicly accessible platforms, we comprehensively analyzed SETD4 gene expression, methylation patterns, and prognostic significance. Furthermore, we investigated its association with cancer‐related pathways, the immune microenvironment, immunotherapy markers, and drug resistance signatures of chemotherapy. Additionally, qRT‐PCR was performed to validate SETD4 expression in clinical specimens.

**Results:**

The expression of SETD4 was abnormal across a variety of cancer types and the expression of SETD4 in colorectal cancer tissues was verified in clinical specimens. The upregulation of SETD4 may be a prognostic risk factor predicting poor overall survival and progression‐free survival. The analysis revealed that the mRNA level of SETD4 was modulated by promoter methylation, and patients with lower methylation levels showed shorter survival times. Pathway analysis showed that SETD4 influenced several key cell cycle pathways, including the G2M checkpoint, and mitotic spindle pathways. In addition, SETD4 negatively affects immune cell infiltration in most cancers, including B cells, CD8 T cells, and macrophages. The correlation between SETD4 and cancer stemness as well as homologous recombination deficiency varied across tumor types, suggesting that SETD4 may play a multifaceted role in tumor resistance. Notably, we identified several potential agents targeting SETD4.

**Conclusions:**

This study demonstrates that SETD4 is an immune‐oncogenic molecule in multiple cancers, with the potential to be a diagnosis, prognosis, and targeted therapy marker.

## Graphical abstract

SETD4 holds great potential as a novel therapeutic target for certain malignancies, and the synergistic manipulation of its related pathways along with immunotherapy or chemotherapy may be clinically valuable in cancer treatment.

## 1 Introduction

Although the mechanisms underlying cancer onset and progression have been extensively researched, malignancies remain a leading cause of mortality globally, with an increasing mortality and incidence trend [1]. Carcinogenesis, the process by which normal cells become cancerous, may be a factor in cancer morbidity and mortality. It is a sophisticated multifactorial process that arises from the accumulation of genomic changes and epigenetic changes [2, 3]. In spite of significant endeavors to enhance cancer diagnosis and treatment over the past few years, clinical outcomes and 5‐year survival rates (SRs) have remained suboptimal [4, 5, 6].

The tumor microenvironment (TME) is a complex system created by interactions among the tumor cells, surrounding tissues, and the immune cells. The TME is crucially involved in the proliferation, migration, and immune evasion mechanisms of tumor cells, ultimately leading to cancer onset [7, 8]. In this regard, patients with an antitumor immune microenvironment characterized by increased T‐cell infiltration often respond better to immunotherapy, especially when treated with immune checkpoint inhibitors [9]. There is growing evidence that tumor mutation burden (TMB) and microsatellite instability (MSI) can serve as molecular indicators of immunotherapy effectiveness [10, 11]. Furthermore, neoantigens have shown great promise for immunotherapeutic applications [12]. However, the emergence of immune evasion and stemness characteristics in tumor cells, caused by alterations in gene expression, epigenetic modifications, and biological pathways, poses a formidable challenge to cancer therapy success [13, 14].

It was in 1994 that cancer stem cells (CSCs) were identified in leukemia [15, 16]. Notably, the limited efficacy of traditional cancer treatment methods against certain malignant tumors could be attributed, at least in part, to the presence of CSCs. These CSCs show characteristics such as metastasis, recurrence, heterogeneity, chemotherapy and radiotherapy resistance, and immunological surveillance evasion [17, 18]. Furthermore, CSCs, given their ability to remain in the G0 phase of the cell cycle and generate new tumors, could drive cancer relapse, metastasis, and Multidrug Resistance (MDR) [19]. The targeting of CSCs may prove to be an efficient method of improving the outcomes of cancer treatment.

As a histone methyltransferase, SET Domain‐Containing Protein 4 (SETD4) is considered to be a critical regulator of cell proliferation, differentiation, and inflammatory responses across various cell lines [20, 21, 22]. It is also crucially involved in stem cell quiescence maintenance via H4K20me3 catalysis facilitation and the subsequent promotion of heterochromatin formation in breast cancer (BC) [23]. Additionally, previous research revealed that SETD4 regulated cell quiescence in CSCs and contributed to drug resistance [24]. Furthermore, RNA interference experiments suggest that downregulation of SETD4 may lead to a greater sensitivity to sorafenib in the HepG2 hepatocellular carcinoma (HCC) cell line, and have an impact on the survival of DU145 androgen‐independent prostate cancer cells [25, 26]. Although SETD4 has recently been implicated in cancer progression and drug resistance, the underlying mechanisms have yet to be unraveled, and a comprehensive pan‐cancer analysis is essential.

This study explored SETD4 gene expression and epigenetic modifications, as well as the protein's implications for prognosis, immunotherapy, and chemotherapy. Furthermore, SETD4 expression was assessed in relation to cancer‐associated pathways, the immune microenvironment, immunotherapy markers, tumor stemness, and genomic instability. The role of SETD4 in various cancers was also elucidated through the construction of co‐expression and coregulatory networks, highlighting its significance as a driver gene in multiple tumor types, as well as the need for additional relevant investigations.

## 2 Materials and Methods

### 2.1 Data Collection

Gene expression, somatic mutation, 450k methylation arrays, and clinical information on 33 malignancies were obtained from The Cancer Genome Atlas (TCGA) (https://portal.gdc.cancer.gov/) using the TCGAbiolinks R package [27]. Corresponding proteome data were procured from The Cancer Proteome Atlas (TCPA) database (https://www.tcpaportal.org/tcpa/) [28], and the Human Protein Atlas (HPA) (https://www.proteinatlas.org/search/SETD4) [29] was used to measure SETD4 protein expression in some tumors. Additional normal tissue sample information was retrieved from the Genotype‐Tissue Expression (GTEx) database [30] using the UCSC Xena platform (https://xenabrowser.net/) [31].

### 2.2 Differential SETD Expression Analysis

Our analysis of SETD4 expression in TCGA cohorts was carried out using the Wilcoxon rank sum test. Because of the limited availability of normal samples, only 24 malignancies were included in the analysis. As a result, the GTEx data set was incorporated to increase the sample size of healthy tissues, and samples were matched to corresponding cancers based on tissue type. Before comparison, batch effects between the two databases were reduced using the limma R package [32]. The pathological stages of patients in TCGA cohorts were determined based on clinical data, and various stages of SETD4 tumor expression were examined using an analysis of variance (ANOVA) test.

### 2.3 Confirming the Hazard Ratio (HR) and the Prognostic Value

A single‐factor Cox regression analysis was performed to assess SETD4's HR on overall survival (OS) and progression‐free survival (PFS) in order to determine the prognostic potential of SETD4. Furthermore, SETD4 was identified as a risk factor in cancers with HR > 1, whereas HR < 1 indicated a protective factor. Based on the median SETD4 mRNA levels of tumor samples from the TCGA cohorts, two expression groups were distinguished: high and low. Subsequently, the Kaplan–Meier (K–M) method was used to analyze OS and PFS outcomes in high and low groups utilizing the survminer R package.

### 2.4 Dna Methylation and Epigenetic Regulation Analysis

Comparison of methylation levels in the promoter regions of tumors and normal samples was performed using Wilcoxon rank sum tests. An analysis of Spearman's correlation test was carried out in order to assess the relationship between DNA methylation and gene expression. Additionally, the prognostic significance of SETD4 methylation was assessed by dividing patients into high‐ and low‐methylated groups based on median methylation levels. As a follow‐up, a K–M survival analysis was performed in order to determine OS and PFS.

### 2.5 Cancer‐Related Pathways and Enrichment Analysis

Gene set enrichment analysis (GSEA) was implemented in the MsigDB database using the cluster Profiler R package [33, 34]. Ten significant cancer‐related pathways identified by Akbani were also included in the analysis [35]. Pathway activity was evaluated via gene set variation analysis (GSVA) [36] on the TCPA data set. The Spearman coefficient was used as a tool to determine the correlation between the levels of SETD4 expression and cancer‐related pathways.

### 2.6 Co‐Expression and Coregulatory Analysis

First, 20 genes that showed the highest correlation with SETD4 were obtained from the GeneMANIA database [37]. An analysis of co‐expression between these 20 genes and SETD4 in 33 TCGA cancer samples was conducted using the Spearman correlation test. Then, Spearman correlation coefficients were determined for SETD4‐associated genes and the activity of 10 prominent cancer‐related pathways. Finally, for each cancer type, individual SETD4 co‐expression and coregulatory networks were constructed based on correlations with a significance level of *p* < 0.01.

### 2.7 Estimation of the Tumor Immune Microenvironment

In 32 solid tumors, the immune microenvironment was explored using the IOBR R package [38]. The EPIC computation algorithm was utilized for assessing immune cell infiltration [39]. The ESTMATE algorithm was utilized to calculate stromal and immune scores [40], and the IPS algorithm was adopted to determine immunophenoscores [41]. These immunological characteristics and SETD4 expression were analyzed by Spearman correlation analysis.

### 2.8 Analysis of Relevance Between Gene Expression and Immunotherapy

The TMB was explored using the maftools R package [42] based on the data from TCGA. Meanwhile, MSI status was assessed using MANTIS software [43] based on Bonneville's study results [44]. An analysis of Spearman correlation was performed on SETD4 expression and these biomarkers. Subsequently, seven immunotherapy cohorts were gathered from public resources, including six data sets (GSE78220 [45], GSE91061 [46], GSE115821 [47], GSE126044 [48], GSE165278 [49], and GSE176307 [50]) from the GEO database and one data set from the IMvigor210 cohort [51]. The Wilcoxon rank sum test was used to make a comparison of SETD4 expression between the response and nonresponse groups. Furthermore, the predicted neoantigen load was obtained from Thorsson's study [52], and further analysis of SETD4 expression correlated with neoantigen load was conducted.

### 2.9 Analysis of SETD4 Relationships With Tumor Stemness and Genomic Instability

mRNA expression‐based mRNAsi and DNA methylation‐based mDNAsi were obtained from Malta's study [53]. On the other hand, data on the predicted HRD score were obtained from Knijnenburg's study [54], along with loss of heterozygosity (LOH), telomeric allelic imbalance (TAI), and large‐scale state transition (LST). An exploratory Spearman correlation analysis was performed to determine whether SETD4 expression correlates with these features/markers.

### 2.10 Evaluating the Influence of Gene Expression on Drug Sensitivity

First, we extracted the experimentally verified drug sensitivity (50% Inhibitory Concentration [IC50]) data from two databases using the oncoPredict R package [55]. The first database was the Genomics of Drug Sensitivity in Cancer (GDSC) database [56], which comprises GDSC1 (including 970 cell lines and 403 compounds) and GDSC2 (including 969 cell lines and 297 compounds). The second database was the Cancer Therapeutics Response Portal (CTRP) [57], and its latest version, CTRPv2, which contains 860 cell lines and 481 compounds, was selected. Using data from these two databases, the Spearman correlation test was utilized to evaluate the influence of SETD4 expression on drug sensitivity, and the false discovery rate was ascertained using the Benjamini–Hochberg method.

### 2.11 Quantitative Real‐Time PCR (qRT‐PCR)

Thirteen samples of colorectal cancer and their corresponding normal tissues were collected from thirteen patients with colorectal cancer who underwent surgical operations at the Guangdong Second Provincial General Hospital. Ethics approval was obtained from the Institution's Ethics Committee (Approval No: 2024‐KY‐KZ‐012‐02). Each patient signed an informed consent form. RNA was extracted with TRIzol reagent (TaKaRa, Japan). Reverse transcription to complementary DNA (cDNA) was performed using HiScript III RT SuperMix (Vazyme, China). The levels of mRNA expression were assessed by qRT‐PCR. The primers sequences of SETD4 are as follows: F:AACATGGCCAAGGAGAGAGC; R: AACATGGCCAAGGAGAGAGC. Fold changes were calculated by normalizing the expression levels to GAPDH in each sample.

### 2.12 Statistical Analysis

Differential expression analysis was conducted using the one‐way ANOVA test and the Wilcoxon rank sum test in multiple groups and between two groups, respectively. The HR was determined through single‐factor Cox regression analysis, and K–M survival curve analysis was conducted using the log‐rank test. An analysis of correlation was carried out using the Spearman correlation coefficient. The statistical analyses were performed using R software (version 4.3.1). Results with *p* < 0.05 were considered to be statistically significant.

## 3 Results

### 3.1 The SETD4 Expression Profile in Different Human Cancers and Pathological Stages

**Figure 1:**
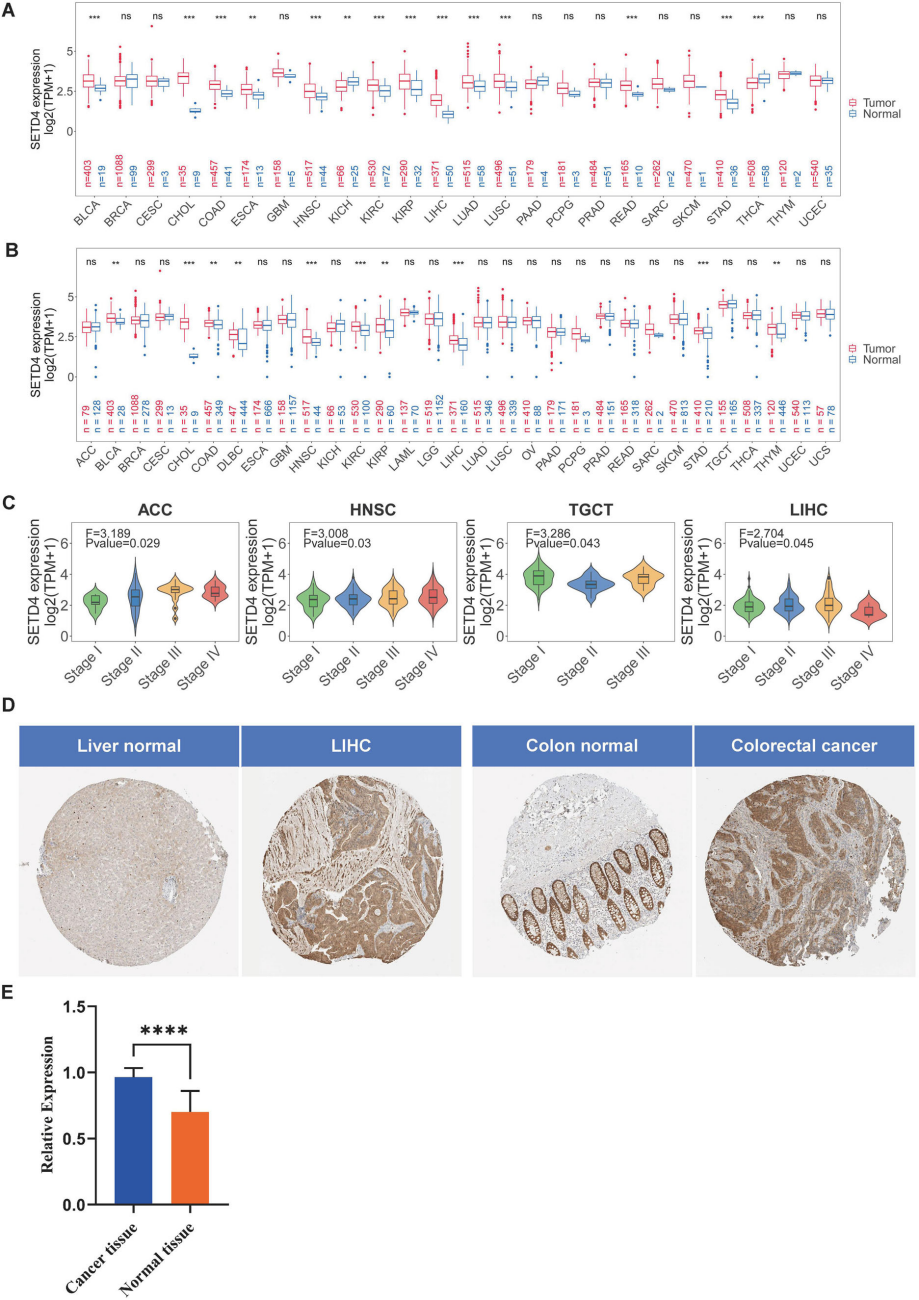
Expression of SETD4 in different kinds of cancer and their corresponding pathological stages. (A) Comparison of SETD4 expression levels in tumor and normal tissues in TCGA cohorts. (B) Comparison of SETD4 expression levels in tumor and normal tissues in combined TCGA and GTEx cohorts. (C) Violin plots displaying the differential SETD4 expression levels at different pathological stages (I, II, III, and IV). Cancers with statistically significant differences in pathological stages are presented. (D) Levels of SETD4 expression in LIHC and colorectal cancer from the HPA database. (E) Levels of SETD4 expression between tumor and normal tissues in patients with colorectal cancer. (*p < 0.05, **p < 0.01, ***p < 0.001, ****p < 0.0001).

Within the TCGA cohort, SETD4 mRNA levels were analyzed in tumor and normal tissues to investigate the effect of SETD4 on various cancers (Figure 1A). Based on the results, SETD4 was upregulated in multiple human malignancies, including bladder urothelial carcinoma (BLCA), lung adenocarcinoma (LUAD), cholangiocarcinoma (CHOL), head and neck squamous cell carcinoma (HNSC), esophageal carcinoma (ESCA), colon adenocarcinoma (COAD), kidney renal clear cell carcinoma (KIRC), liver hepatocellular carcinoma (LIHC), lung squamous cell carcinoma (LUSC), kidney renal papillary cell carcinoma (KIRP), rectum adenocarcinoma (READ), and stomach adenocarcinoma (STAD). Conversely, SETD4 downregulation was observed in thyroid carcinoma (THCA) and kidney chromophobe (KICH). As TCGA cohorts include a limited number of normal samples, healthy tissues from GETx were incorporated along with the corresponding cancer types for further comparative analysis. According to the results, SETD4 was still upregulated in most of the cancers, such as BLCA, COAD, HNSC, and STAD, and downregulated in KICH and THCA (Figure 1B). Notably, lymphoid neoplasm Diffuse Large B‐cell lymphoma (DLBC) and Thymoma (THYM) showed a heightened SETD4 expression when additional normal samples were included in the analysis (Figure 1B). Subsequently, we assessed SETD4 expression across various pathological stages, revealing significant variations across adrenocortical carcinoma (ACC), HNSC, testicular germ cell tumors (TGCT), and LIHC. Moreover, SETD4 mRNA levels showed an upward trajectory in advanced pathological stages (Figure 1C). According to the HPA database, a high level of SETD4 expression was observed in LIHC and colorectal cancer (Figure 1D). To further elucidate the disparity in the SETD4's expression between tumor and normal tissues, a qRT‐PCR study was performed on 13 human colorectal cancer cases and their corresponding normal samples. Results showed that a significant overexpression of SETD4 mRNA was observed in colorectal cancer cells compared to normal tissues (Figure 1E).

### 3.2 Prognostic Value of SETD4 mRNA Expression Throughout Pan‐Cancer

**Figure 2:**
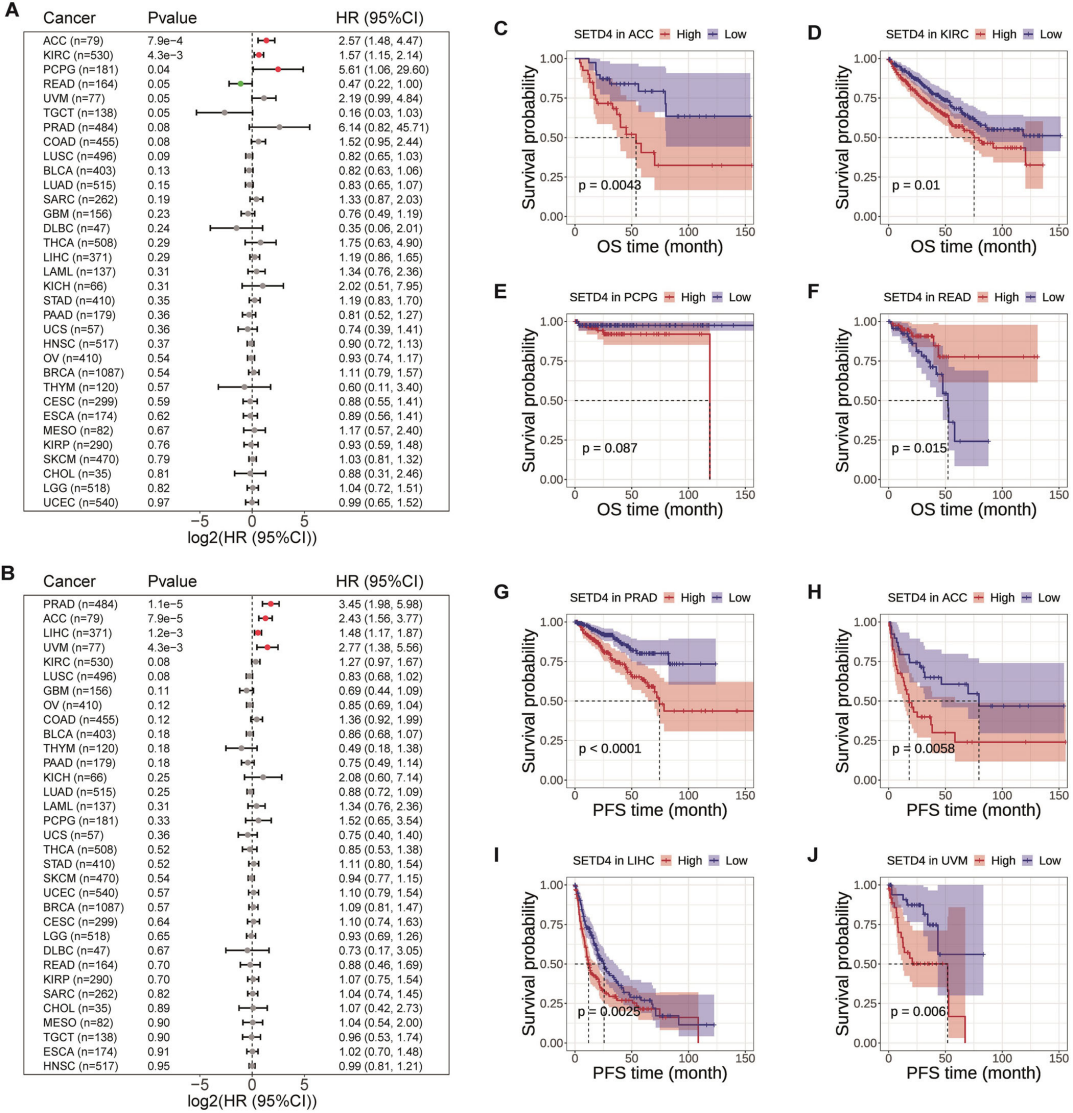
HR and prognostic value of SETD4 in OS and PFS. (A) Analysis of SETD4 in OS using the Cox regression model. (B) Analysis of SETD4 in PFS using the Cox regression model. (C) OS differences in ACC between groups with high and low SETD4 expression levels. (D) OS differences in KIRC between groups with high and low SETD4 expression levels. (E) OS differences in PCPG between groups with high and low SETD4 expression levels. (F) OS differences in READ between groups with high and low SETD4 expression levels. (G) PFS differences in PRAD between groups with high and low SETD4 expression levels. (H) PFS differences in ACC between groups with high and low SETD4 expression levels. (I) PFS differences in LIHC between groups with high and low SETD4 expression levels. (J) PFS differences in UVM between groups with high and low SETD4 expression levels.

According to the notable SETD4 upregulation observed in the aforementioned various types of cancers, we examined the influence of SETD4 on cancer patients. Specifically, we conducted an analysis on TCGA cohorts to determine the HR for OS and PFS based on SETD4 expression. Based on the results, there was a positive correlation between SETD4 expression and poor OS in ACC, KIRC, and pheochromocytoma and paraganglioma (PCPG). Conversely, SETD4 upregulation exerted a protective effect in READ (Figure 2A). Moreover, SETD4 expression correlated positively with the likelihood of disease progression in prostate adenocarcinoma (PRAD), ACC, LIHC, and uveal melanoma (UVM) (Figure 2B). Furthermore, we evaluated the prognostic influence of SETD4 on tumor development and recurrence across various cancer types by using K–M survival analysis (Figure 2C–J). According to the results, high SETD4 expression was associated with poorer OS in ACC (Figure 2C) and KIRC patients (Figure 2D) and worse PFS in PRAD (Figure 2G), ACC (Figure 2H), LIHC (Figure 2I), and UVM patients (Figure 2J). Overall, SETD4 may function as an oncogene in specific cancer types, with its overexpression potentially contributing to disease progression.

### 3.3 Epigenetic Modification Alternations of SETD4 in Cancer Cohorts

**Figure 3:**
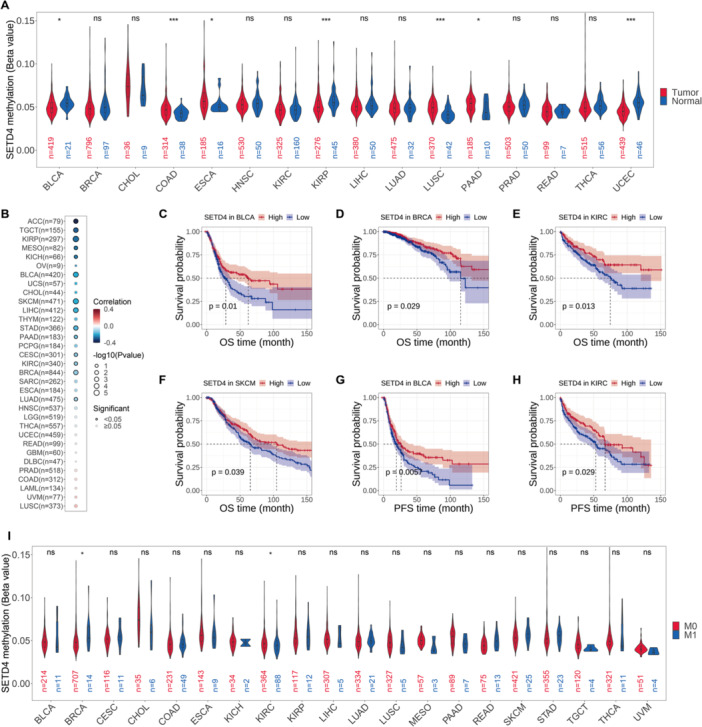
Alterations in SETD4's DNA methylation and its corresponding prognostic value. (A) Methylation levels of SETD4 between tumor and normal samples in TCGA cohorts. (B) Correlation between promoter methylation and mRNA expression of SETD4 in various human cancers. (C) Prognosis of SETD4 methylation on OS in BLCA. (D) Kaplan–Meier curves showing the prognosis of SETD4 methylation on OS in BRCA. (E) Prognosis of SETD4 methylation on OS in KIRC. (F) Prognosis of SETD4 methylation on OS in SKCM. (G) Prognosis of SETD4 methylation on PFS in BLCA. (H) Prognosis of SETD4 methylation on PFS in KIRC. (I) SETD4 methylation levels between tumors in M0 and M1 stages. The M0 stage represents primary tumors and the M1 stage represents metastatic tumors. (*p < 0.05, ***p < 0.001).

According to the promoter methylation status analysis conducted on tumor and normal samples, SETD4 was hypomethylated in both (Figure 3A). Furthermore, SETD4 hypomethylation was significantly high in BLCA, KIRP, and uterine corpus endometrial carcinoma (UCEC), with COAD, ESCA, LUSC, and pancreatic adenocarcinoma (PAAD) showing even higher levels (Figure 3A). Additionally, promoter methylation and SETD4 gene expression were negatively correlated, particularly in ACC, TGCT, KIRP, mesothelioma (MESO), and KICH (Figure 3B). Based on these findings, we delved deeper into the prognostic values of SETD4 methylation in various cancers. Lower methylation levels were correlated with poorer OS in BLCA (Figure 3C), BRCA (Figure 3D), KIRC (Figure 3E), and skin cutaneous melanoma (SKCM) (Figure 3F) and worse PFS in BLCA (Figure 3G) and KIRC (Figure 3H). On further comparing the methylation level of SETD4 in primary (M0 stage) and metastasized tumors (M1 stage), we found that patients with metastasis showed higher methylation levels in BRCA, whereas decreased methylation levels were found in KIRC. SETD4 methylation levels did not differ significantly among other cancer types (Figure 3I); perhaps because there were fewer samples at the M1 stage, a trend of hypomethylation in SETD4 was still discernible in CHOL, MESO, PAAD, TGCT, and UVM. These findings collectively suggest that epigenetic methylation in multiple cancer types modulated SETD4 gene expression and that some cancers may benefit from SETD4 promoter methylation as a prognostic indicator.

### 3.4 Cancer‐Related Pathways and SETD4 Expression

**Figure 4:**
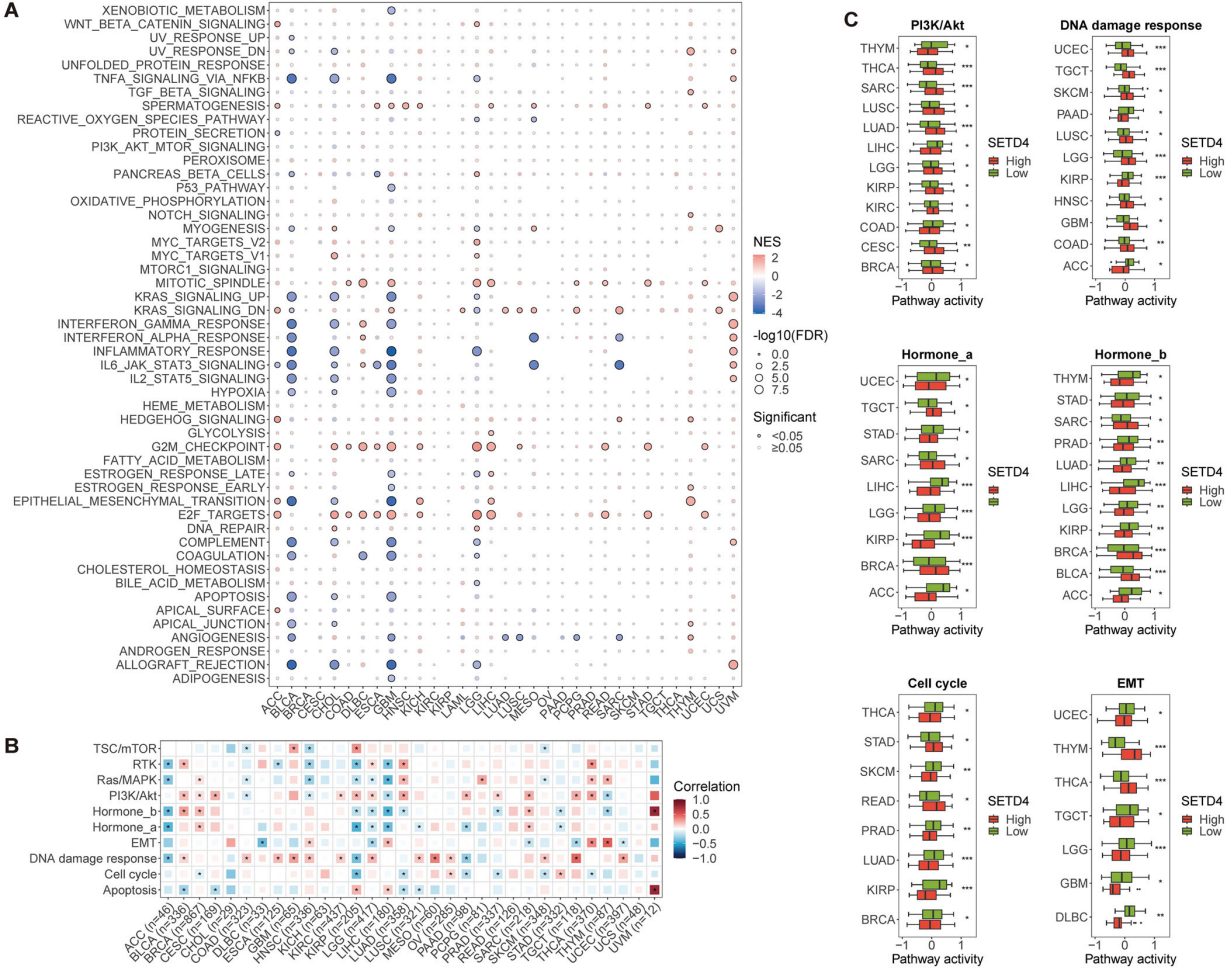
Correlation between SETD4 expression and pathway activity. (A) GSEA for hallmark cancer signaling pathways between high‐ and low‐expression SETD4 patients. (B) Correlation between SETD4 expression and 10 salient cancer‐related pathways. Pathway activity was calculated by GSVA. (C) Comparison of the DNA damage response, PI3K/Akt pathway, Hormone_a, Hormone_b, cell cycle, and the EMT pathway across cancers. Cancers with statistically significant differences in pathological stages are presented. (*p < 0.05, **p < 0.01, ***p < 0.001).

As part of the study, we performed GSEA to evaluate the relationship between SETD4 mRNA levels and hallmark pathways' activity within TCGA data sets and to explore the potential functions of SETD4 in tumorigenesis. According to the results, SETD4 expression correlated positively with various cell cycle–related pathways (such as the G2M checkpoint and mitotic spindle pathways) across different cancer types (Figure 4A). Further analysis was carried out for cancers in which patients having higher SETD4 expression presented a poorer prognosis. Based on the results, it was found that SETD4 exerted a notable positive impact on various pathways, particularly in ACC, LIHC, and UVM (Figure 4A). Specifically, in ACC, the upregulation of the Wnt/β‐catenin signaling, hedgehog signaling, G2M checkpoint, epithelial–mesenchymal transition (EMT), and E2F target pathways were observed with high SETD4 expression. Additionally, in LIHC, individuals with higher SETD4 expression levels showed G2M checkpoint, E2F targets, and spindle pathway activation. Furthermore, UVM patients with SETD4 upregulation showed heightened activity in various signaling pathways, including IL6/JAK/STAT3 signaling, IL2/STAT5 signaling, and allograft rejection pathways. Conversely, there was a negative correlation between the expression of SETD4 and immune‐related pathways [such as TNFA signaling via NFKB, KRAS signaling upregulated, and Interferon Gamma (IFN‐γ) response] in BLCA, CHOL, and glioblastoma multiform (GBM) (Figure 4A). Another interesting finding was that SETD4 was positively correlated with the EMT pathway in ACC, KICH, UVM, and THYM, suggesting a potential role of SETD4 in cancer metastasis (Figure 4A). We further conducted a detailed examination of the impact of SETD4 on the 10 prominent cancer‐related pathways using data from the TCPA cohort. According to the results, SETD4 primarily activated the DNA damage response (13/32) and PI3K/Akt (12/32) pathways and tended to inhibit the Hormone_a (7/32), Hormone_b (8/32), and cell cycle (7/32) pathways (Figure 4B). Additionally, the effects of SETD4 on the EMT pathway showed opposite patterns across different cancers, with activation in HNSC, LIHC, THCA, and THYM and inhibition in DLBC, LGG, SARC, TGCT, and UCEC (Figure 4B). A comparative analysis of the activity levels of these six pathways between the SETD4 high‐ and low‐expression groups revealed that SETD4 expression correlated positively with DNA damage response and PI3K/Akt pathway activity and negatively with Hormone_a, Hormone_b, cell cycle, and EMT pathway activity across various cancers (Figure 4C). Based on these findings, SETD4 can be recognized as a crucial player in the pathogenesis and progression of cancer.

### 3.5 Co‐Expression and Co‐Regulatory Networks of SETD4

**Figure 5:**
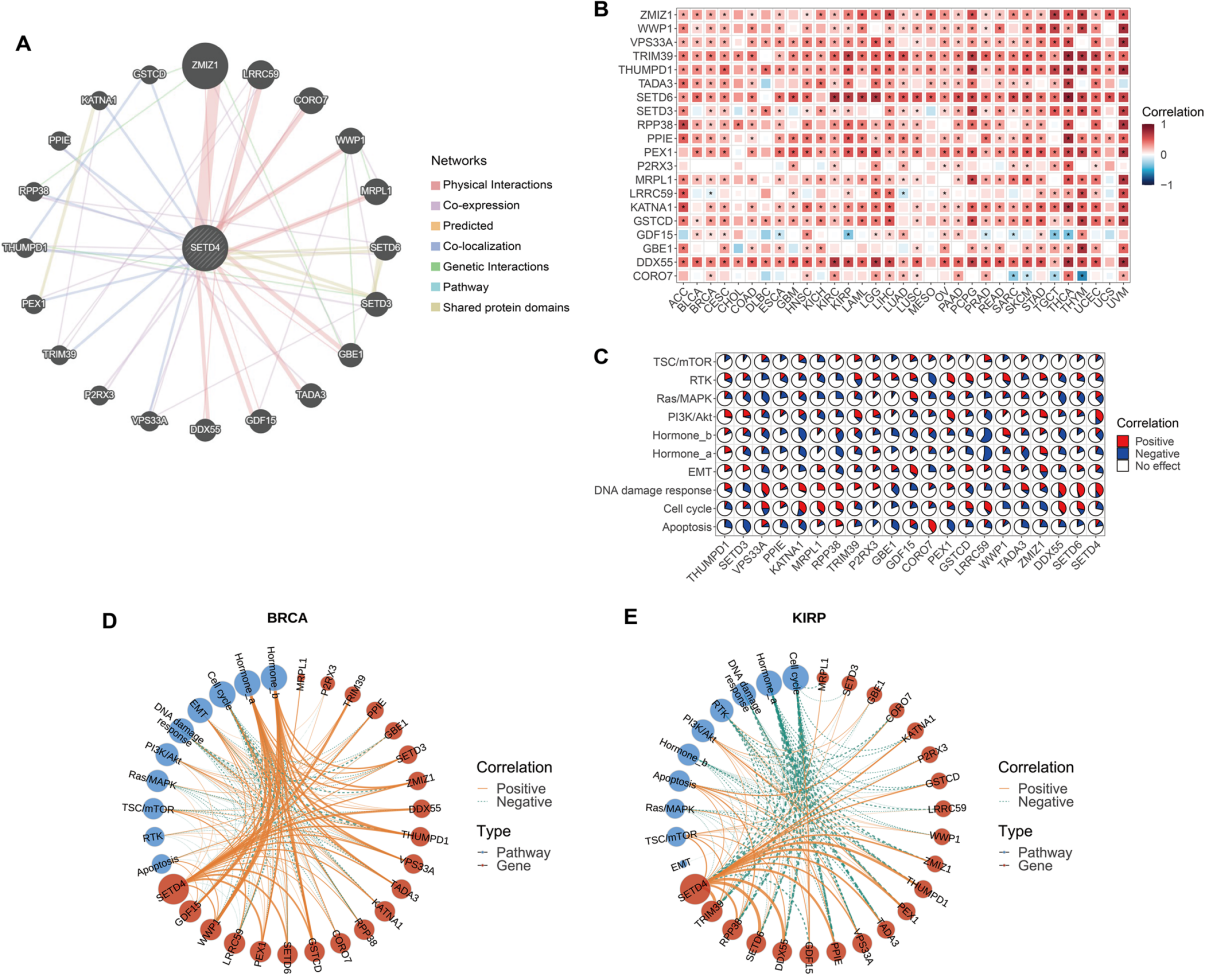
Co‐expression and coregulatory network of SETD4. (A) Interaction network of 20 genes most correlated to SETD4 provided by GeneMANIA. (B) Correlation on mRNA between the correlated genes and SETD4 in TCGA cohorts. (C) Pie plots showing the percentage of pathways regulated by correlated genes and SETD4 in each cancer. (D) Co‐expression and coregulatory network of SETD4 in BRCA. (E) Co‐expression and coregulatory network of SETD4 in KIRP.

First, 20 genes (THUMPD1, SETD3, VPS33A, PPIE, KATNA1, MRPL1, RPP38, TRIM39, P2RX3, GBE1, GDF15, CORO7, PEX1, GSTCD, LRRC59, WWP1, TADA3, ZMIZ1, DDX55, and SETD6) from the GeneMANIA database that showed the highest correlation with SETD4 were identified (Figure 5A). The genes that interacted were then used to reassess associations with SETD4 at the mRNA level within the TCGA cohorts, revealing significant co‐expression patterns (Figure 5B). The impact of these genes on various pathways was further evaluated to establish their involvement in cancer‐related pathways at the protein level. According to the results, these genes regulate various cancer‐related pathways extensively, including Hormone_a, Hormone_b, DNA damage response, and cell cycle pathways (Figure 5C). Otherwise, we found varied impacts between SETD4‐related genes on the EMT pathway in various cancers. Among these genes, GDF15 has been confirmed to play a metastasis‐promoting role in most studies [58, 59, 60], whereas ZMIZ1 has been found to affect invasion and metastasis in tongue squamous cell carcinoma (TSCC) [61]. Moreover, the overexpression of WWP1 in ICC cells induced the proliferation of cells and increased their ability to metastasize [62]. These findings revealed the complex regulatory network of SETD4 and its related genes on EMT, and highlighted their potential role in tumor metastasis once again. We then constructed a SETD4‐centric co‐expression and coregulatory network specific to each cancer type using the co‐expression patterns of SETD4 and its correlated genes, along with their regulatory mechanisms within cancer‐related pathways. Notably, these genes were implicated in Hormone_a and Hormone_b pathway activation in BRCA (Figure 5D). Conversely, the genes inhibited the activity of both pathways in KIRP (Figure 5E). These findings indicate the distinct functional roles of the identified genes in human malignancies.

### 3.6 The Correlation of SETD4 With the Tumor Immune Microenvironment

**Figure 6:**
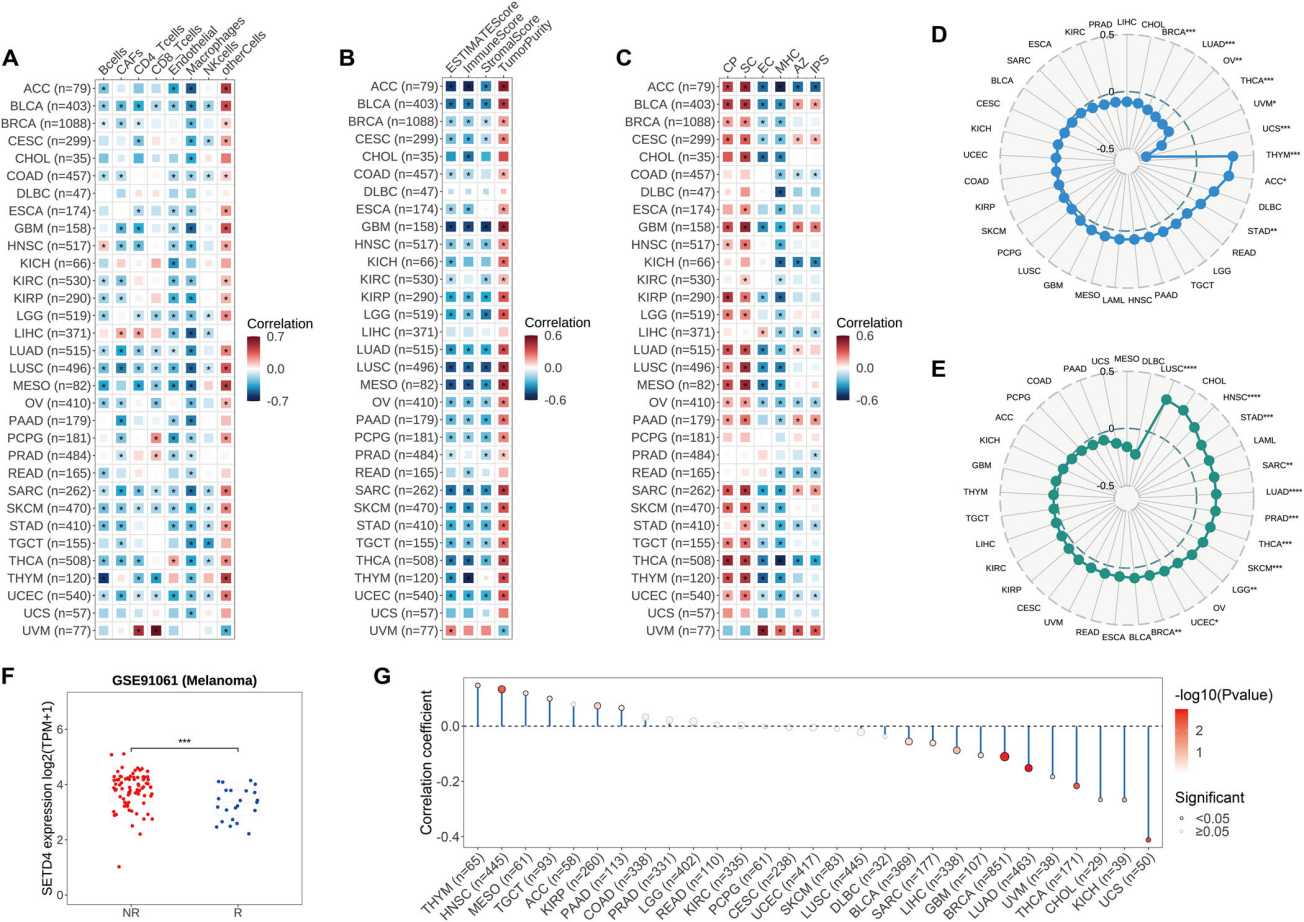
Correlation of SETD4 with the tumor immune microenvironment and immunotherapy markers. (A) Association of SETD4 expression with immune cell infiltration in TCGA solid tumors. (B) Association of SETD4 expression with stromal and immune scores in TCGA solid tumors. (C) Association of SETD4 expression with immunophenoscores in TCGA solid tumors. (D) Correlation of SETD4 with TMB. (E) Correlation of SETD4 with MSI. (F) In melanoma patients, SETD4 mRNA levels were significantly lower following treatment with immune checkpoint inhibitors. (G) Correlation between SETD4 expression and neoantigen burden. (*p < 0.05, **p < 0.01, ***p < 0.001, ****p < 0.0001).

Herein, we found a strong relationship between SETD4 expression and immune‐related pathways in various cancers. It was necessary to investigate further the role of SETD4 in the immune microenvironment of 32 solid tumors in order to understand this phenomenon. First, the EPIC algorithm was used to evaluate the immune cell infiltration level, and the subsequent analysis showed a notable negative correlation of SETD4 expression with B cell (18/32), Cancer Associated Fibroblast (CAF; 19/32), CD4 T cell (15/32), CD8 T cell (10/32), endothelial cell (21/32), macrophage (29/32), and NK cell (12/32) count in most cancer types (Figure 6A). This finding suggests the potential carcinogenic involvement of SETD4 in promoting an immunosuppressive TME. Notably, due to the negative correlations observed between SETD4 and various immune cells in some cancer types, BLCA, LUSC, Sarcoma (SARC), SKCM, and UCEC patients with lower SETD4 levels may benefit from immunotherapy. Furthermore, the ESTIMATE algorithm was performed to predict stromal and immune cell scores, revealing a consistent inverse relationship between SETD4 mRNA levels and comprehensive ESTIMATE immune scores (26/32), as well as immune scores (25/32) and stromal scores (22/32) across multiple cancer types (Figure 6B). The findings indicate a positive relationship between SETD4 expression and tumor purity, further confirming the potential correlation between high SETD4 expression and lower immune cell infiltration, which could explain immunotherapy failure. Previous research has demonstrated the utility of the IPS in predicting responses to immune checkpoint inhibitors. In this regard, we explored the interplay between SETD4 expression and IPS to assess the former's impact on immunotherapeutic outcomes [41]. According to the results, SETD4 correlated negatively with the major histocompatibility complex (MHC) and effector cells (ECs) in most cancer types and positively with suppressor cells (SCs) and immune checkpoints (CPs) (Figure 6C). Consequently, patients with high SETD4 expression were more immunogenic but less responsive to immunotherapy. Nonetheless, there was limited concordance between SETD4 expression and IPS scores. Specifically, SETD4 correlated positively with immune checkpoint inhibitors in some cancer types, including BLCA, CESC, PAAD, SARC, and UVM, but negatively in ACC, COAD, KICH, LIHC, Ovarian Serous Cystadenocarcinoma (OV), READ, STAD, THCA, and UCEC (Figure 6C). Our analysis also revealed a relationship between SETD4 expression and immunotherapy markers, such as TMB and MSI. Specifically, SETD4 correlated positively with TMB in THYM, ACC, and STAD, but negatively in UCS, UVM, THCA, OV, LUAD, and BRCA (Figure 6D). Additionally, SETD4 correlated significantly positively with MSI in several cancer types, including UCS, HNSC, STAD, SARC, LUAD, PRAD, THCA, SKCM, LGG, UCEC, and BRCA (Figure 6E). Given these robust associations between SETD4 and immunotherapy markers, we speculated that SETD4 could distinguish immunotherapy responses. However, we tested a series of seven immunotherapy cohorts and found that SETD4 expression was significantly lower in responsive patients in a melanoma data set (Figure 6F). Furthermore, we examined the condition of neoantigens in diverse cancers and found that SETD4 expression correlated significantly positively with neoantigens in THYM, HNSC, MESO, TGCT, KIRP, and TAAD and negatively in UCS, KICH, CHOL, THCA, UVM, LUAD, BRCA, GBM, LIHC, SARC, and BLCA (Figure 6G). These findings indicate potential intricate interactions between SETD4 expression and the immune microenvironment, implying that some cancer patients with low SETD4 expression might benefit from immunotherapy.

### 3.7 SETD4 Expression and Cancer Chemotherapy

**Figure 7:**
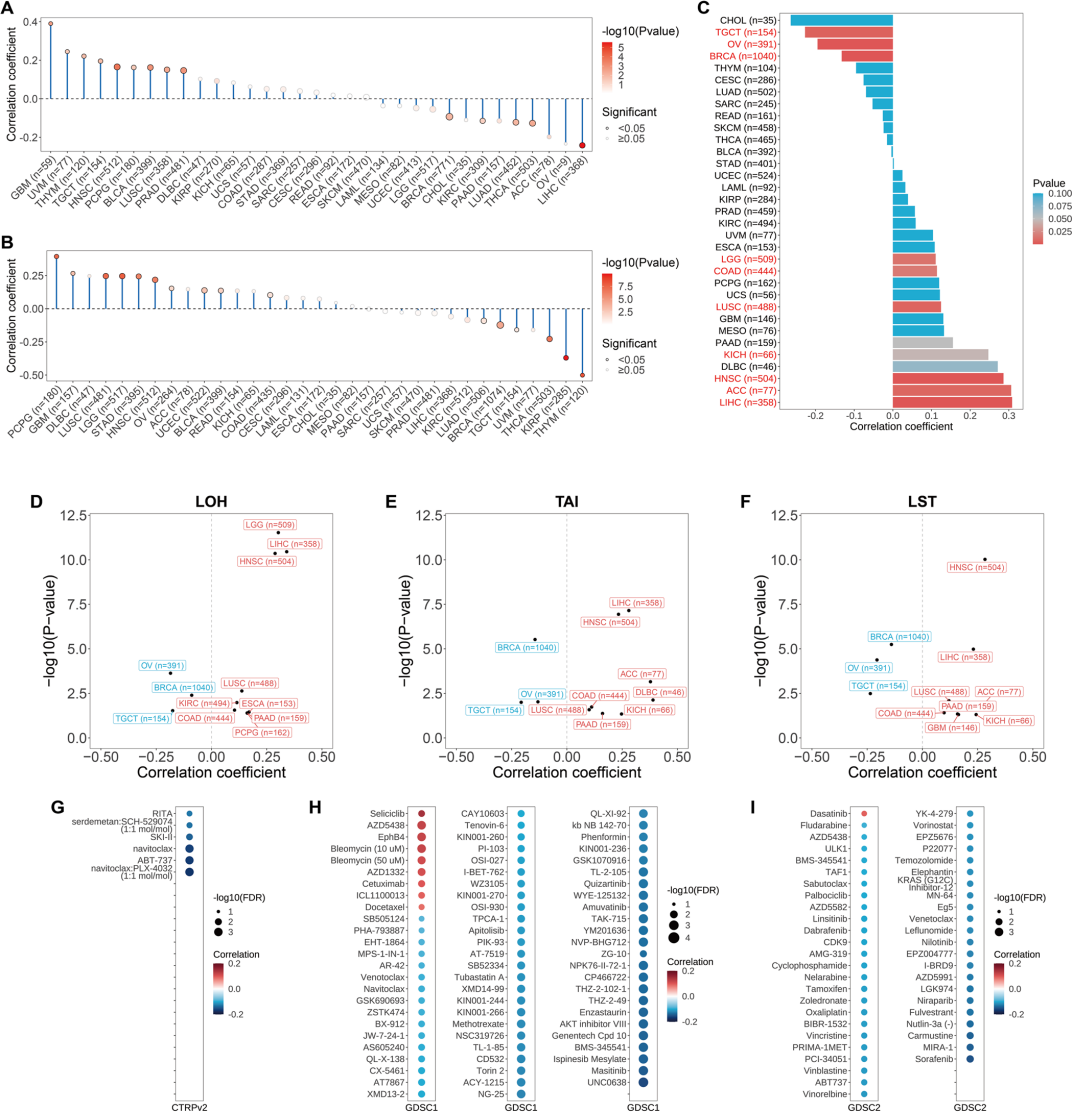
Association of SETD4 expression with the stemness indices and HRD and drug sensitivity. (A) Relationship between SETD4 and the cancer cell stemness score (mRNAsi) in TCGA cancers. (B) Relationship between SETD4 and the cancer cell stemness score (mDNAsi) in TCGA cancers. (C) Relationship between SETD4 and HRD. Cancer types with significantly statistical correlation are in red. (D) Correlation of SETD4 expression with LOH in diverse cancers. (E) Correlation of SETD4 with TAI in diverse cancers. (F) Correlation of SETD4 with LST in diverse cancers. (G) Relationship between SETD4 and the sensitivity of drugs in CTRPv2. (H) Relationship between SETD4 and the sensitivity of drugs in GDSC1. (I) Relationship between SETD4 expression and the sensitivity of drugs in GDSC2.

Previous studies have shown the notable participation of stem‐like cells in cancer advancement and chemotherapy resistance [14, 19]. Herein, we used mRNA expression and DNA methylation data to examine the relationship between SETD4 and tumor stemness indicators (specifically mRNAsi and mDNAsi). Based on the results, SETD4 expression correlated significantly positively with mRNAsi in PCPG, GBM, DLBC, LUSC, and LGG and negatively in THYM, KIRP, THCA, UVM, and TGCT (Figure 7A). On the other hand, SETD4 correlated significantly positively with mDNAsi in GBM, UVM, THYM, TGCT, and HNSC and significantly negatively in LIHC, ACC, THCA, LUAD, and PAAD (Figure 7B). These findings suggest that patients with elevated mRNAsi or mDNAsi levels may show increased tumor stemness and drug resistance, with SETD4 potentially serving as a tumor stemness marker in some cancer types. Additionally, it is well known that HRD can impair DNA repair mechanisms, inducing genomic instability [63], thus rendering HRD‐positive cancers susceptible to platinum‐based chemotherapeutic agents and PARP inhibitors [64]. As a result, we examined the correlation between SETD4 expression and HRD, revealing significant associations in a few cancer types. Specifically, the expression of SETD4 was positively correlated with the HRD score in LIHC, ACC, HNSC, DLBC, and KICH and negatively in CHOL, TGCT, and OV (Figure 7C). The link between SETD4 expression and HRD‐induced genomic instability markers [LOH (Figure 7D), TAI (Figure 7E), and LST (Figure 7F)] was further analyzed, revealing consistent patterns with HRD, thus implying a potential link between SETD4 and genomic instability in cancer. As a result, we deduced that LIHC, ACC, HNSC, DLBC, and KICH patients with elevated SETD4 levels may benefit from treatment with platinum‐based chemotherapeutic drugs or PARP inhibitors. Using data from the GDSC (Figure 7G) and CTRP [GDSC1 (Figure 7H), GDSC2 (Figure 7I)] databases, a drug sensitivity analysis was further conducted to evaluate the influence of SETD4 on the responsiveness of malignant tumors to pharmacological interventions. Intriguingly, few drugs showed a positive association between IC50 values and SETD4 expression, implying that cells with SETD4 upregulation were resistant to a select few drugs, including seliciclib, AZD5438, EphB4, bleomycin, AZD1332, cetuximab, ICL1100013, docetaxel, and dasatinib, and showed heightened sensitivity to numerous others. Despite SETD4's propensity to induce increased tumor stemness and drug resistance, the availability of a greater variety of targeted drugs for chemotherapy is a promising development.

## 4 Discussion

The SET domain‐containing proteins (SETDs) are histone lysine methyltransferases that methylate histone proteins to regulate chromatin structure and gene expression [65, 66]. According to research, SETD4, a member of SETDs, is involved in CSC quiescence and drug resistance in BC and NSCLC [23, 24]. Furthermore, high SETD4 expression is correlated with a lack of Estrogen Receptor (ER) expression in BC [20]. Given SETD4's potential involvement in carcinogenesis, cancer advancement, and drug resistance, there is a pressing need to delve deeper into its significance in biological processes as well as its potential clinical relevance in cancer onset, progression, and treatment.

Herein, we demonstrated the oncogenic role of SETD4 in various TCGA cancer types. Integration of TCGA and GTEx data revealed SETD4 mRNA upregulation in BLCA, COAD, HNSC, KIRC, KIRP, LIHC, and STAD. Subsequent analysis showed a correlation between SETD4 upregulation and advanced pathological stages in ACC, HNSC, TGCT, and LIHC. These findings suggest a potential association of SETD4 expression with tumor progression, and invasion, emphasizing the potential application of SETD4 as a biomarker for cancer detection and surveillance. As a result, the HR of SETD4 was calculated for OS and PFS in various cancer types, revealing that SETD4 upregulation poses a risk for poor OS in ACC, KIRC, and PCPG and a risk for worse PFS in PRAD, ACC, LIHC, and UVM. We also evaluated the prognostic significance of SETD4 in the aforementioned malignancies, and thus carried out a survival analysis. Consistent with our hypothesis, patients with high SETD4 expression showed poorer OS in ACC and KIRC and worse PFS in PRAD, ACC, LIHC, and UVM. These discoveries emphasize the importance of SETD4 as a prognostic indicator in cancer. Hitherto, the restricted research has suggested the potential participation of SETD4 in the pathogenesis of diverse cancers, including BC [20, 23], NSCLC [23], and prostate cancer [26]. Furthermore, the precise mechanisms of SETD4 in both normal physiological processes and disease progression remain unclear. Our pan‐cancer GSEA yielded significant insights into the association between SETD4 expression and critical signaling pathways in cancer. Specifically, we discovered a correlation between SETD4 upregulation and the activation of cell cycle‐related pathways in various cancers, suggesting the potential involvement of SETD4 in cancer cell proliferation. Intriguingly, SETD4 expression correlated negatively with malignancy and immune‐related pathways including KRAS signaling, immune response, and IL2/STAT5 signaling pathways in BLCA, CHOL, and GBM, and positively in UVM.

In the last few decades, immunotherapy has greatly impacted the treatment of cancer in a variety of ways, with immune checkpoint inhibitors demonstrating sustained therapeutic impacts [67]. However, the effectiveness of these treatments is restricted to particular subsets of cancer patients. Immune cell infiltration in the TME, a crucial factor in tumor progression, has demonstrated a significant impact on immunotherapy outcomes [9]. Herein, we elucidated the influence of SETD4 on immune‐related pathways in various cancers, presenting it as a promising biomarker for immunotherapeutic interventions. Additional correlation analysis revealed that SETD4 correlated negatively with the infiltration of different immune cell types (including B cells, macrophages, and NK cells) in most cancer types. Conversely, SETD4 correlated positively with activated CD4 and CD8 T cells in UVM. Subsequent IPS analysis confirmed these findings, revealing that SETD4 expression correlated consistently negatively with both EC and MHC and positively with CP and SC. Notably, compared to other tumor types, UVM showed contrasting results. These findings indicate that in addition to predominantly contributing to immunosuppressive microenvironment development in most cases, SETD4 may also exert an immune‐activation effect in UVM. In this regard, we speculated that SETD4 could serve as a predictive marker for patients' clinical response to immune checkpoint blockade, a hypothesis that we tested across various immunotherapy cohorts. Among a cohort of patients with advanced melanoma undergoing anti‐CTLA4 and anti‐PD1 therapy, the nonresponsive subgroup showed notable SETD4 upregulation. This finding suggests that SETD4 may exert a detrimental influence on the tumor immune microenvironment, emphasizing its potential as a novel immune target. Therefore, concurrent administration of anti‐SETD4 antibodies along with immune checkpoint inhibitors could be a promising anticancer therapeutic approach.

As reported earlier, CSCs could be promising targets for cancer therapy. According to research, SETD4 plays a role in the quiescence of CSCs and drug resistance regulation in BRCA and NSCLC [23, 24]. Additionally, SETD4 inhibition could enhance the sensitivity of the HepG2 HCC cell line to sorafenib [25]. Our analysis also revealed that SETD4 was significantly positively correlated with mRNA‐expression–based and DNA‐methylation–based stemness indices in GBM, HNSC, PCPG, and LUSC. However, these two indices showed opposite characteristics in UVM, THYM, and TGCT. Moreover, the examination of the link between SETD4 and drug sensitivity across various cell lines from GDSC and CTRP revealed that SETD4 upregulation was associated with resistance to certain drugs. Nonetheless, alternative pharmacotherapies may serve as better strategies.

This study had several limitations. First, the bioinformatics findings require validation through additional experimental research. A comprehensive inquiry into the relationship between SETD4 and cancer‐related pathways is also needed. Furthermore, the epigenetic regulation of SETD4 and its influence on cancer cell stemness should be explored further. Additionally, our observation that cells with SETD4 upregulation showed heightened drug sensitivity in most cases contradicts prior research, necessitating validation through additional experiments.

## 5 Conclusions

Herein, we thoroughly explored the gene expression profile and prognostic significance of SETD4, as well as its associations with the immune microenvironment and cancer‐related pathways in TCGA data sets. Our discoveries highlight the significance of SETD4 in predicting prognosis, therapeutic responses, and drug sensitivity through intricate regulatory mechanisms involving immune cell infiltration, immune‐related pathway modulation, tumor cell stemness maintenance, and homologous recombination pathway regulation. The oncogenic characteristics of SETD4 render it a promising option for immunotherapeutic and chemotherapeutic interferences. Nonetheless, systematic investigations into the role of SETD4 in cell cycle regulation, tumor stemness, and related pathways are required to further elucidate its contribution to tumor resistance against immunotherapeutic and chemotherapeutic agents. Overall, SETD4 holds great potential for use as a novel therapeutic target for certain malignancies, and the synergistic manipulation of its related pathways along with immunotherapy or chemotherapy may be clinically valuable in cancer treatment.

## Author Contributions

Conceptualization: Yuyun Zhong and Liyue Sun. Data curation: Bin Peng. Formal analysis: Ruiqi Wang, Zijie Huang, Zhaoting Hu, and Bin Peng. Funding acquisition: Yuyun Zhong and Ruiqi Wang. Investigation: Zijie Huang. Methodology: Yuyun Zhong. Project administration: Yuyun Zhong and Bin Chen. Resources: Ruiqi Wang. Validation: Zijie Huang and Bin Chen. Writing–original draft: Zhaoting Hu. Writing–review and editing: Liyue Sun.

## Ethics Statement

The Clinical Ethics Committee of Guangdong Second Provincial General Hospital (No: 2024‐KY‐KZ‐012‐02).

## Conflicts of Interest

The authors declare no conflicts of interest.

## Data Availability

The data sets that were used and/or analyzed during the present study are obtainable from the corresponding author upon reasonable request.
